# Experiences with the quality of telemedical care in an offshore setting – a qualitative study

**DOI:** 10.1186/s12913-023-09664-5

**Published:** 2023-06-20

**Authors:** Michael Stefan Hellfritz, Alexander Waschkau, Jost Steinhäuser

**Affiliations:** Universität zu Lübeck, Institute of Family Medicine, University Medical Center Schleswig-Holstein, Campus Lübeck, Ratzeburger Allee 160, 23562 Lübeck, Germany

**Keywords:** Telemedicine, Remote consultation, Quality of health care, Oceans and seas, Offshore, Wind energy

## Abstract

**Background:**

The evaluation and the improvement of the quality of telemedical care become increasingly important in times where this type of care is offered to a broad number of patients more and more. As telemedical care in an offshore setting has already been in use for decades, analyzing the extensive experience of offshore paramedics using telemedical care can help identify determinants of quality. Therefore, the aim of this study was to explore determinants of the quality of telemedical care using the experiences of experienced offshore paramedics.

**Methods:**

We conducted a qualitative analysis of 22 semi-structured interviews with experienced offshore paramedics. The results were categorized in a hierarchical category system using content analysis as described by Mayring.

**Results:**

All 22 participants were males, having a mean of 3.9 years of experience working with telemedicine support offshore. Generally, participants stated that for them telemedical interaction did not differ much from personal interaction. However, the offshore paramedics personality and way to communicate were mentioned to impact the quality of telemedical care as it influenced the way cases were presented. Furthermore, interviewees described it to be impossible to use telemedicine in cases of an emergency as it was too time-consuming, technically too complex, and lead to cognitive overload as other tasks with higher priority needed their attention. Three determinants of a successful consultation were mentioned: low levels of complexity in the reason for consultation, telemedical guidance training for the teleconsultant physician and for the delegatee.

**Conclusion:**

Appropriate indications for telemedical consultation, communication training of consultation partners, and the impact of personality need to be addressed to enhance the quality of future telemedical care.

**Supplementary Information:**

The online version contains supplementary material available at 10.1186/s12913-023-09664-5.

## Background

Initially, the utilization of telemedicine has been restricted to niche remote sectors such as the offshore sector and was not available in routine medical health care [1–3]. Meanwhile, also due to the COVID pandemic, telemedicine acceptance in general has grown and it has shown to be effective [4–6]. However, studies still show low acceptance of and reservations against the widespread utilization of telemedicine amongst policymakers and communities [7–10]. Therefore, measuring and improving the quality of telemedical care becomes more important.

In high-seas and offshore industries, the provision of health care via telemedicine has a decade-long history already, with thousands of telemedical consultations being performed every year [11–13]. Therefore, the experiences of offshore users of telemedicine may help identify generalizable determinants for the quality of telemedical care.

In the German North Sea, offshore wind workers are already provided with telemedical care on a large scale for years [13, 14]. Over the course of the last ten years, the offshore paramedics have gained fundamental and extensive experience in the use of telemedicine.

The objective of this study was to identify determinants of quality for telemedical care offshore by exploring the experiences of offshore paramedics regarding the quality of the telemedical care they provide.

## Methods

### Study design

This study followed a qualitative approach. This approach allows the reconstruction of the reality of the interviewees and reduces the impact of the researchers onto the findings.

Between March and Mai 2020 MH conducted semi-structured one-to-one interviews with experienced offshore paramedics who work on offshore structures in the German North Sea. This study complied with the Consolidated Criteria for Reporting Qualitative Research (COREQ) [15]. The COREQ-Checklist is available in the supplement material.

### Setting

The interviewees were all German paramedics with additional professional training. German paramedics have a three-year emergency technician level training. Their primary expertise is the assistance of the emergency physician in the stabilization of emergency patients and the transport to an appropriate hospital. All interviewees received additional training especially in the handling of Standard Operating Procedures (SOP), that allow medical procedures outside their original core expertise, e.g. medication administration. They are deployed and stationed offshore without a physician or another medical professional present and are assisted by laymen only who have received a basic first-aid training. They are equipped with an onboard hospital, advanced medical equipment and telemedical devices. The offshore paramedics are trained to work autonomously within the boundaries of SOPs for the most common medical cases offshore. For advanced treatment, the treatment of cases outside of SOPs, and the decision to evacuate a patient, they perform a synchronous telemedical consultation with hospital-based onshore teleconsultant physicians.

The teleconsultant physicians are based onshore in the emergency departments of hospitals with maximum care capacity. Teleconsultant physicians answer consultation calls in their work time and in addition to their regular duties in the emergency department. They usually are physicians in postgraduate training to become a clinical specialist. Telemedical consultation can result in two scenario: The patient is treated offshore or the patient is evacuated via helicopter or other means of transportation, depending on the seriousness of the case [13].

### Sample

Interviewees were recruited via e-mail invitation or during presentations by MH in the weekly videoconference of a dispatching medical company following a purposeful sampling strategy. They were informed about methods, aims, and the former deployment of MH as an offshore paramedic. Inclusion criteria for participation in the interviews were being non-physician medical personnel that operates telemedicine and is experienced in the use of offshore telemedicine. The interviewees did not receive any compensation.

### Procedure

The interviews followed a semi-structured interview protocol consisting of 10 guiding questions, developed by JS and MH. The interview protocol was reevaluated after three interviews, no changes were made. No repeat interviews were carried out. The guiding questions are available in the supplement material. The interviews were conducted by MH in person (1), via telephone (15) or videoconference (6). All interviews followed the same approach, the interviewees were encouraged to elaborate on the topics to their liking.

### Researchers

MH is a male medical student and former employee of the dispatching medical company with experience in offshore telemedicine and knew eight of the interviewees personally, prior to the study. AW is a male psychologist and scientific researcher with extensive experience in qualitative and telemedicine research. JS is a male academic GP and professor of family medicine with extensive experience in qualitative and telemedicine research.

### Analysis

Inductive and deductive qualitative content analyses as proposed by Mayring [16] were carried out by two reviewers separately (AW, MH). This methodology allows a systematic and yet open and flexible analysis of the content and therefore reduces bias of researchers.

The interviews were audio recorded, transcribed verbatim and pseudonymized. No additional notes were taken, the interviewees did not comment on transcripts or findings. For this, both reviewers read the transcripts independently, identified key quotes of the interviewees and developed a hierarchical category system of findings with major and minor categories as well as codes with individual quotations. The category system was organized along the three dimensions of quality established by Donabedian: structure, process and outcome quality [17]. Structure quality describes material, personnel, and administrative conditions that medical care is delivered in. Process quality maps the immediate interaction between the physician, the medical assistants, the administrators, and the patient. And outcome quality describes the outcome of the medical condition of the patient. One additional main category was created. A final consensus version was developed in subsequent consensus meetings with all authors.

## Results

25 individuals were invited to participate in the interviews, 22 accepted. All participants were male German paramedics who work offshore, were between 26 and 52 years old, and had 2 to 7 years of experience with offshore telemedicine. The duration of the interview was between 17 and 57 min. Additional sociodemographic data can be found in Table 1.

**Table 1:** Sociodemographic data

Characteristics			
Gender	male: 22 (100%)		
profession	paramedic: 22 (100%)		
	mean	median	min/max
Year of birth	1983	1985	1994/1968
professional work experience (years)	14.3	13	5/31
offshore medical experience (years)	3.9	4.5	2/7
Duration of interview (minutes)	35	32	17/57

The interviewees generally welcomed the study and interest in the field of work. Data saturation was achieved, as there could no new categories be formed from the last five interviews. The results of the detailed qualitative analysis of the experiences of the interviewees are listed in in Tables 2, 3, 4 and 5.

**Table 2:** Structure Quality

Major Category	Reported Experiences
Connection to internet	Good quality of internet connection benefitted quality of telemedical consultations.
Training of teleconsultant physician	Familiarization of the teleconsultant physician with offshore medical equipment and expertise benefitted quality of telemedical consultations.
	Consultant medical specialties available 24/7 at a hospital with maximum care benefitted quality of telemedical care.
Training of offshore paramedic	Original professional training of offshore paramedics focused on emergency care rather than primary health care.
	The intentional training, prior to deployment, of the particular mindset and associated skills that are required to work as an autonomous offshore paramedic benefitted telemedical care.
	Training of offshore paramedic in autonomous treatment of patients and in Crew Resource Management benefitted quality of telemedical care.
Joint training	Thorough and joint telemedical training and technical induction for teleconsultant physician and offshore paramedic benefitted quality of telemedical consultations.
	Simulation training with utilization of the actual telemedical technology, that is in use on current deployments benefitted quality of telemedical care.

**Table 3:** Process Quality

Major Category	Reported Experiences
Indication for the use of telemedicine	Seeking medical support during treatment of non-acute patients (e.g., primary health care issues, delegation of medication, etc.).
	Creating legal security relating to treatment (ad-hoc and post hoc medical documentation) and medical evacuations.
Time management and cognitive overload	Telemedical consultation in emergencies was possible only after treatment of patients.
	Consultations done by teleconsultant physician simultaneously with other duties limited quality of telemedical consultations.
Medical data protection rules	In cases of emergency, medical data protection rules could not always be adhered to.
Standardization	Default technical settings on a single-button communication line for telemedical consultation and precise standardized organizational authorities for offshore paramedics benefitted quality of telemedical care.
	Standardized indications for consultation and evacuation benefitted quality of telemedical care.
Communication	Eloquence and telemedical leadership abilities of teleconsultant physician benefitted quality of telemedical consultations.
	Treatment of patients and work with assistants without knowledge of a shared language limited the quality of telemedical consultations.
	Linguistic “sender-receiver-problems” during consultations limited quality of telemedical consultations.
	Communication with transferal hospital prior to arrival of patient benefitted quality of telemedical care.
Strengths of telemedicine	One patient – multiple physicians concept benefitted quality of telemedical care.
	Professional relationships between teleconsultant physician and offshore paramedic benefitted from telemedical consultations.
Limitations	Loss of situational information due to shortened duration of conversation and impossibility of tactile feedback for teleconsultant physician limited the quality of telemedical consultations.
	Relationship-building between teleconsultant physician and offshore paramedic as well as patient was a challenge.

**Table 4:** Outcome Quality

Major Category	Reported Experiences
Offshore paramedics’ comparison of offshore and onshore treatment	There was no difference between direct and telemedical consultations and treatment.
	Use of telemedicine reduced transports of patients to hospital.

**Table 5:** Offshore Paramedic

Major Category	Reported Experiences
Impact of personality	Offshore paramedics influenced the course of treatment willingly and unwillingly by directive narration.
	Offshore paramedics reconsulted with physicians they personally knew in case of disagreement with teleconsultant physician.
	Offshore paramedic treated patients without consultation of teleconsultant physician.
Offshore paramedic as mediator	Eloquence of offshore paramedic influenced the course of treatment of patients.
	Mutual trust and good fit between offshore paramedic and teleconsultant physician benefitted telemedical consultations.
	Treatments occurred with no direct contact between teleconsultant physician and patient offshore.

### Structure quality

The majority of the consultations were reported to be related to non-emergency cases. As trained paramedics specialized in emergency medical care, primary health care did not fall within the offshore paramedics’ field of expertise. Therefore, telemedicine served as a facilitator of primary health care competence.

For me personally, telemedicine provides me with a certain degree of security in primary health care and in areas we are not trained in in depth, and which require specialist knowledge. TN22

Interviewees pointed out, that the training and mindset of German paramedics, in principle, aimed at the arrival and physical presence of an emergency physician. Their original task was to assist in the emergency treatment of patients. The scenario offshore with a teleconsultant physician, however, required a different mindset and workflow. This mindset and the required skills should be trained for intentionally prior to the deployment of the offshore paramedic.

We all have our mindset and our methods to work through a medical emergency. But at a certain point, an emergency physician always arrives, if need be. […] It makes a big difference whether this emergency physician physically enters the room or whether I put on a headset and there is a physician at the other end of the line. This is something we could have trained for. TN12

Additionally, it was pointed out that the professional qualification of the teleconsultant physician was essential to the quality of the consultations. Our participants reported good experiences with hospitals that offer maximum care because they have all necessary specializations at hand. At the same time, interviewees felt that evacuations were premature. They reported a conflict of interest between the offshore paramedic and the teleconsultant physicians. Offshore paramedics wished to diagnose and treat as many patients as possible offshore to avoid unnecessary evacuations, while teleconsultant physicians were seeking best possible diagnosis and treatment as well as legal security for their therapeutic decisions. This was experienced as an inhibiting factor to the use of telemedicine.

We, in principle, would like to keep patients offshore and prevent evacuations. The physicians, however, focus on legal security and treatment of patients, of course. But in case of doubt, they tend to evacuate immediately. In some cases, this might be premature. This hinders the utilization of telemedicine. TN2

### Process quality

Indication for the use of telemedicine

Interviewees defined three main areas of medical indication for the use of telemedicine: seeking medical support, creating legal security for themselves, and consulting about medical evacuations.

Interviewees reported that they mainly used telemedicine to seek synchronous medical support in the broad field of primary health care issues.

For us, it [telemedicine] is very helpful, especially in primary health care. TN7.

Additionally, telemedicine was used to have a teleconsultant physician clear a pharmacological therapy or delegate other advanced procedures outside the core expertise of the offshore paramedics.

…to receive delegation for a procedure or medication that was not cleared originally… TN20.

Interviewees reported that they used telemedicine to create additional legal security for themselves, even in situations in which they would have felt comfortable assessing and treating the patient by themselves. They consulted telemedically about recommendations for or procedures on patients synchronously as well as retrospectively to the treatment of patients.

Offshore, I need to do telemedical consultation for legal protection, even if I don’t actually need it. TN4

Interviewees stated that they regularly used telemedicine to consult teleconsultant physicians to get a recommendation regarding medical evacuations. The decision to evacuate a patient to an onshore medical facility was described as difficult with various and cost-intensive consequences. According to interviewees, their purpose on board was to prevent any unnecessary costly evacuations and therefore work stoppage and delays offshore. They stated that non-medical considerations had an influence on the decisions about medical evacuations and that other responsible personnel offshore should be included in the decision process via telemedical consultations. This would result in a shared responsibility of all stakeholders offshore for this difficult decision.

…each MedEvac needs to be delegated, recommended by the physician at the least. […] This is why telemedicine is great for us, because this way, responsibility does not lie entirely on your own shoulders. TN8

Some paramedics were unclear about their liability in a telemedical care setting. They felt unsure about their own accountability when treating a patient and used telemedicine to document consultations for their own legal protection. The difference between delegated and recommended medical procedures and the resulting legal implications were not always clear to them.

…who is responsible in the end? Is it the physician or is it me? Do I carry implementation responsibility only? Who is responsible if something goes wrong? In my opinion, there is still some insecurity. The same is true for documentation… TN4

#### Cognitive overload and time management

The Participants emphasized that in cases of emergencies, telemedicine could only be used after the primary treatment and stabilization of the emergency patient. During primary treatment telemedical consultations were impossible because they were time-consuming, technically too complex and required mental capacity that is needed for emergency medical procedures. Interviewees usually were assisted by unskilled laymen only and needed to tend to patients at the same time as consulting with a teleconsultant physician. In these scenarios of cognitive overload, interviewees reported to prioritize the treatment of the patient over the consultation of a superior physician.

I am too occupied on site to be able to communicate with the doc. I have two laymen assisting me who, at best, I have trained myself for a few weeks. At worst, they are on their first day and don’t even know the rescue bag and I have to do everything by myself. TN12

Teleconsultant physicians were reported to often have to consult in addition and simultaneously to their regular duties in an onshore hospital. This let to sub-optimal consultations with the teleconsultant physician being rushed to premature decisions and the offshore paramedic having the feeling of not getting the appropriate consultation.

They [teleconsultant physicians] themselves are in a rush and start to lose a lot of time needed for their original tasks. […] Teleconsultant physicians should be able to concentrate solely on you and should not need to answer additional consultations at the same time. TN9

### Outcome quality

With regards to outcome quality, statements focused on comparing the treatment of patients onshore and offshore. Some interviewees did not see any differences at all in the medical care and interaction between patient and physician when comparing offshore and onshore. They defined three major determinants of a successful consultation: the survey needs to be easy to apply, the teleconsultant physician needs to be trained to instruct well, and the delegatee needs to be trained for the procedure.

If the bandwidth is sufficient, the physician-patient dialogue can be conducted in the same way as in person. I don’t see a big difference. TN2

Participants commented controversially about patients’ satisfaction with telemedical care. Some reported that patients were generally satisfied with their treatment and welcomed the possibility of additional expertise, while others reported that patients were concerned about a possible substandard treatment by an offshore paramedic.

You are “only” being treated by the paramedic, the nurse, the EMT…TN14.

### The offshore paramedic

All participants in this study were German trained paramedics who received additional non-standardized training to qualify for offshore telemedical care of both, emergency and non-emergency patients.

#### Mediation via telemedicine

Participants reported that during consultations patients and teleconsultant physicians did not necessarily have immediate interaction. Interviewees felt that they acted as mediator between the two parties, as sensory organ for the teleconsultant physician, and as translator for the patient. This moderation of the consultation was reported to require mutual trust in the individual, the training, and the situational handling of the two consulting parties. This did not always work out to the satisfaction of the two parties.

There is no direct contact to the patient. With the telemedical equipment, I mediate the reality to the physician. TN20

And all this because, in cases where we use telemedicine and the paramedic acts as a sort of middleman, the physician has to trust the information given by the paramedic. Some are better at this than others. TN13

#### Impact of personality on telemedical care

Several interviewees reported that they felt to be a key participant in the consultation and that they had major influence on the resulting procedure and treatment of the patient, both willingly and unwillingly. They said it was challenging for them to objectively present the case to the teleconsultant physician. All recommendations of teleconsultant physicians would mainly depend on the way the offshore paramedics described the case rather than on objective medical information, they said. Some interviewees even reported that they intentionally used these possibilities to get delegations from the teleconsultant physician, which would only confirm their own preformed treatment plan. This way of working was reported to mainly focus on the decision whether or not an evacuation was necessary. Another interviewee reported that, in case where he disagreed with the teleconsultant physician’s assessment, he would simply contact a different physician he knows via alternative ways of communication to get a different recommendation that would fit his own concept.

Do I want the patient to be evacuated? If yes, I tell the story exactly how it happened because the physician will then want to evacuate. If I want the patient to stay offshore, honestly, I choose my words in a way that the physician will not deem an evacuation necessary. TN3

## Discussion

In this study, telemedical consultation offshore was described as best practicable in non-emergency situations and the majority of telemedical consultations were reported to be related to such cases.

The aspect that it seems impossible to use telemedicine in cases of emergency stands in opposition to the proposed concept of telemedicine as a facilitator for emergency care in remote (offshore) environments [18, 19]. An improvement of technical devices and organizational aspects might increase the confidence of users of telemedicine to perform telemedical consultations and might increase utilization in emergency cases offshore [20].

Internationally, the personnel for offshore telemedical care varies greatly [18, 21]. Our interviewees were all professionally trained paramedics with primary training in treatment of emergency patients and the assistance to emergency physicians. However, the reported majority of consultations were related to sub-acute non-emergency patients [13]. Training for, both, emergencies, and primary health care as well as a possible adaptation of personnel to the context of deployment seems desirable.

Our interviewees reported good experiences with teleconsultant physicians working at hospitals that offer maximum care. At the same time, they reported a tendency to premature evacuations due to (alleged) limited possibilities of diagnosis offshore. This might be related to the fact, that the teleconsultant physician works in an environment with a high incidence of serious diseases. Therefore, General Practitioners (GP) should be considered as teleconsultant physicians too [22]. GPs are specialized in continuous, non-disruptive treatment of sub-acute primary health care, based on the concept of watchful waiting and the detection of any red flags with limited possibilities of diagnosis [23]. Furthermore, onshore telemedical consultation of German GPs can resolve most cases conclusively already [24].

Interviewees in our study described that the decision of the teleconsultant physician to evacuate a patient seemed to depend more on the way the case was presented, then the objective medical information provided. This might indicate an insufficient level of standardization in the communication. Studies have shown that different health professions each have their own linguistic communication style and that ineffective communication between the partners can negatively affect health care outcomes [25, 26]. Miscommunication between the teleconsulting partners already has been identified as one of the most-common preventable risks to patient safety [25–31]. Additionally, communication training can alter and improve handling and treatment of primary health care patients even via telemedical consultation [32–34]. Other effects of the increasing utilization of telemedical care on patient safety are not yet adequately identified and analyzed and literature lacks recommendations on how to address patient safety in the training of telemedical personnel [35, 36].

### Strengths and Limitations

Most of the existing literature discusses the quality of telemedical care offshore without a solid data base [37]. This study offers rare insights into the extensive first-hand experiences and therefore can be a valuable basis for further analysis of the quality of telemedical care offshore. The results of this study can be used to form hypotheses for further research.

One author, MH, is an experienced offshore paramedic himself who has profound understanding of the situational circumstances, strengths, and limitations in which telemedical care offshore is performed. He therefore was able to profoundly comprehend the experiences of the participants. His familiarity with eight of the participants helped create an interview environment, in which uneasy experiences could be reported.

We are aware that this study has several limitations. This study did not analyze the patients’ experiences nor their satisfaction with telemedical care offshore, an essential part of the analysis of the quality of care [17]. Furthermore, no teleconsultant physicians were interviewed. Future studies should focus on the experiences patients and teleconsultant physicians have had with telemedical care offshore.

All participants in this study have the same employer and follow the same concept of telemedical care offshore. This might indicate a selection bias in the findings.

## Conclusion

Our interviewees reported that telemedical consultation is not feasible in emergency situations, but rather in primary health care cases. Therefore, General Practitioners should be considered as teleconsultant physicians too. Communication training and standardization might benefit outcome quality of telemedical care offshore.

## Electronic supplementary material

Below is the link to the electronic supplementary material.

Supplementary Material 1

Supplementary Material 2

## Data Availability

The datasets generated and analyzed during the current study are not publicly available due to the specifications of the ethics committee approval. For requests, please contact michael.hellfritz@student.uni-luebeck.de.
