# Supplementary material for: Experiences with the quality of telemedical care in an offshore setting – a qualitative study
Source: BMC Health Serv Res. 2023 Jun 20;23:661. doi: 10.1186/s12913-023-09664-5 (PMC10283170; doi:10.1186/s12913-023-09664-5)
Supplement: Supplementary file 1 — Supplementary Material 1 [file 12913_2023_9664_MOESM1_ESM.pdf]

Guiding questions „OffshoreTeleWind“

Warm up

1. What experiences with telemedicine have you had on offshore structures?

Main part

2. What affects the medical care with telemedicine in your experience?
3. What differences have you experienced when comparing onshore and offshore treatment?
4. In what kind of situations was the telemedical equipment sufficient for a safe treatment of the patient offshore?
5. What insecurities did you experience in the context of telemedical care?
6. In what kind of situations would you use different means of communication then the ones, provided by the company? (E. g. WhatsApp, email, phone, etc.)
7. What additional equipment would you want to have for the telemedical care offshore?
8. What differences did you observe in the utilization of various telemedical equipment?
9. What should be changed in the training for utilization of telemedical equipment?

Cool down

10. What aspects are important to you in addition to the ones above?

In this manuscript, we report on findings regarding all questions, except Nr. 7 and Nr. 8.
